# Accuracy and adequacy of photoprotection in pediatric systemic lupus erythematosus patients, and the effect of education on photoprotection: a prospective study

**DOI:** 10.1186/s12969-023-00901-z

**Published:** 2023-10-17

**Authors:** Porntipa Suebsarakam, Dara Mairiang

**Affiliations:** Department of Pediatrics, Faculty of Medicine, Khon Kaen University, Khon Kaen, 40002 Thailand

**Keywords:** Pediatric, Photoprotection, Systemic lupus erythematosus, Ultraviolet, Therapeutic patient education

## Abstract

**Background:**

Systemic lupus erythematosus (SLE) is a systemic autoimmune disease that is associated with multiple organ involvement and leads to significant morbidity and mortality. One of the important environmental factors that influences the exacerbation of preexisting SLE is ultraviolet (UV) radiation, so photoprotection is essential. The aims of this study were to evaluate the accuracy and adequacy of photoprotection in pediatric SLE patients, and to investigate the effect of education on photoprotection.

**Methods:**

SLE patients aged ≤ 18 years who attended pediatric outpatient clinics were prospectively enrolled. The accuracy and adequacy of photoprotection were assessed by the questionnaire, and compared between baseline and the ≥ 3-month follow-up timepoint. Comprehensive written and verbal photoprotection education was provided to all patients and parents/caregivers after the first assessment.

**Results:**

One hundred patients were included (mean age 13.6 ± 2.5, 92% female). At the first assessment, 79% of patients used sunscreen with a sunburn protection factor ≥ 30 (77%) and protection grade of ultraviolet A +  +  + (63%). Fifty-two percent of patients applied sunscreen every day. A minority of patients applied an adequate amount of sunscreen (32%), used water-resistant sunscreen (34%), used lip balm with sunscreen (23%) and reapplied sunscreen when sweating (13%). The most commonly missed areas when applying sunscreen were the ears and dorsum of the feet. The least often practiced sun protection behavior was wearing sunglasses. The most often reported activities during the peak UV index, were playing with friends and walking to the cafeterias. At the second assessment, the majority of photoprotection practices were improved in all aspects except using water-resistant sunscreen, reapplying sunscreen when sweating, applying sunscreen on the ears and dorsum of feet, and wearing sunglasses. The main reason for not using sunscreen switched from thinking it was unnecessary at the first assessment to disliking its texture at the second assessment.

**Conclusions:**

Education on photoprotection was effective in improving photoprotection practices. The photoprotection practices that need to be specifically emphasized are applying an adequate amount of sunscreen and using lip balm with sunscreen. The photoprotection which were least practiced at both the first and seconds assessments were reapplying sunscreen when sweating, applying sunscreen on the ears and dorsum of the feet, and wearing sunglasses.

**Supplementary Information:**

The online version contains supplementary material available at 10.1186/s12969-023-00901-z.

## Introduction

Systemic lupus erythematosus (SLE) is a systemic autoimmune disease that is associated with multiple organ involvement and leads to significant morbidity and mortality. One of the important environmental factors that influences exacerbations of SLE is ultraviolet (UV) radiation [1].

UV radiation induces the release of inflammatory chemokines and cytokines, which in turn recruit inflammatory cells. UV radiation also induces necrosis and apoptosis of keratinocytes, which leads to an accumulation of nucleic acids. When the nucleic acids combine with autoantibodies, plasmacytoid dendritic cells are activated. This leads to the production of interferon-alpha and the activation of auto-reactive T-cells. Interferon-alpha subsequently induces the production of more inflammatory chemokines and cytokines, which causes an amplification cycle of inflammation [2]. Therefore, photoprotection from the sun to reduce UV radiation exposure can effectively prevent a lupus flare, esp. in the cutaneous lupus erythematous, and is an essential component of SLE treatment [3, 4].

Studies conducted in adults with cutaneous lupus erythematosus (CLE), which is a patient population that is particularly sensitive to UV radiation, reported a significant deficiency in the use of photoprotection [5], and only a minority of CLE patients reported consistently wearing sunscreen [6]. A study in adults with SLE found that the majority of patients had good photoprotection awareness. However, they did not translate into better photoprotection practices or better disease activity [7]. These findings are in contrast to those from a previous study that reported that patients who regularly used sunscreen had a better clinical outcome [8].

Effective photoprotection in children requires multiple modalities of patient and parent/caregiver cooperation, which may be difficult to achieve in real-life practice [9]. Children’s behavior is influenced by their age and development. Moreover, children and adolescents have different activities, risk behaviors, and types of recreation compared to adults, and these factors can also increase their exposure to UV radiation [10, 11]. Another important factor is that the effect of UV radiation is more pronounced in children than in adults as children have a lower concentration of protective melanin and a thinner stratum corneum [12].

Biologically active vitamin D is formed in the skin after exposure to UV radiation [13]. Photoprotection can lead to vitamin D deficiency [14] and may contribute to the progression of active SLE disease due to the importance of vitamin D in regulating the immune response [15].

The aims of this study were to evaluate the accuracy and adequacy of photoprotection in pediatric SLE patients, and the effect of education on photoprotection. The secondary objectives were to examine the association between photoprotection and SLE disease activity. and to investigate the effect of photoprotection on serum vitamin D level.

## Methods

### Patients

This prospective study enrolled SLE patients aged ≤ 18 years who attended the pediatric outpatient rheumatology, nephrology, or dermatology clinics of Srinakarin Hospital, Faculty of Medicine, Khon Kaen University, Khon Kaen, Thailand during the October 2020 to September 2022 study period. Patients with an overlapping syndrome, unwilling to participate, incapable of completing the questionnaire, or unable to follow-up were excluded. SLE was diagnosed according to Systemic Lupus International Collaborating Clinics (SLICC) classification criteria [16] by pediatricians who subspecialize in rheumatology, nephrology, or dermatology. The protocol for this study was approved by the Khon Kaen University Ethics Committee for Human Research (COA no. 631392), and complied with all of the principles set forth in the 1964 Declaration of Helsinki and all of its subsequent amendments. Verbal assent and/or written informed consent to participate was obtained from all included study participants and their parent.

### Photoprotection questionnaire

The photoprotection questionnaire was completed primarily by the patient with assistance as needed by the patient’s caregivers and/or the research assistant. The questionnaire was administered in a private room and apart from the patient’s subspecialist. The questionnaire elicited information about the following topics: 1) Characteristics of sunscreen use and the frequency of sunscreen application. 2) The amount of sunscreen used to apply to the face in fingertip unit 3) The area of bodily sunscreen coverage by asking the patient to color the anatomic regions to which they normally apply sunscreen 4) The sun protection habits index (SPHI), which was defined as the average of a 4-point Likert scale of sun protection behaviors ranging from rarely/never (1 point) to always (4 points). The sun protection behaviors included wearing sunscreen, wearing a shirt with sleeves, wearing a hat, staying in the shade or under an umbrella, and wearing sunglasses [17]. 5) Time of day of sun exposure, and the activities that the patient was involved in during times of exposure. The patients were asked to consider their answers in relation to a week of completing the questionnaire. The term “sunscreen use” means any use.

### Disease activity assessment

SLE disease activity was assessed using the Systemic Lupus Erythematosus Disease Activity Index 2000 (SLEDAI-2 K) [18] and Cutaneous Lupus Erythematosus Disease Area and Severity Index (CLASI) [19]. These disease activity assessments were performed at both baseline and at the second follow-up.

### Second assessment

After patients completed the photoprotection questionnaire, they underwent a medical assessment, and written and verbal patient education specific to comprehensive photoprotection was provided by the patients’ subspecialists according to the recommendations published by the American Academy of Dermatology [20, 21], the American Academy of Pediatrics [22], the Dermatological Society of Thailand [23], and the European League Against Rheumatism [24]. Photoprotection habits, disease activity, and serum Vitamin D level were then reassessed at the at least 3-month follow-up, and those results were compared to the baseline results for each measured parameter. Vitamin D was measured in terms of 25-hydroxyvitamin D (25OHD) levels with electrochemiluminescence immunoassay on an automatic Roche Cobas e601 analyzer (Roches Diagnostics, Mannheim, Germany). 25OHD had a measuring range of 3–70 ng/mL.

### Statistical analysis

Patient demographics and clinical characteristics were summarized using descriptive statistics. Parametric data were reported as mean and standard deviation (SD), and compared using paired student *t*-test. Non-parametric data were given as median and interquartile range (IQR), and compared using Mann Whitney U test. McNemar test and Wilcoxon signed-rank test were used to determine differences between the first and second assessments for paired categorical and ordinal data, respectively. The correlation between disease activity and photoprotection index was analyzed using Spearman correlation coefficient. A multivariate logistic regression model was used to determine the effect of the medication doses relative to changes in disease activity and vitamin D level. All data analyses were performed using Statistics SPSS version 19. Statistical significance was set at *p*-value < 0.05.

## Results

### Patients

One hundred and seven patients were initially enrolled. Of those, 3 patients were excluded due to loss to follow-up, 3 patients were transferred to other centers, and 1 patient died before completion of the assessment. In total, 100 patients were included in the study. The baseline demographics of the patients are shown in Table 1.

**Table 1:** Baseline demographic data of the patients

Characteristics	Values
Age, years, mean (SD)	13.6 (2.5)
Female gender (n)	92
Duration of disease, years, median (IQR)	1.8 (0.4–3.9)
Household income, Thai baht/month, median (IQR)	10,000 (5,500–21,500)
Interval between the first and second visit, days, median (IQR)	113 (95–154)
Subspecialty clinic (n)	
- Nephrology clinic	56
- Rheumatology clinic	41
- Dermatology clinic	3
Medications (n)	
- Hydroxychloroquine	97
- Prednisolone	83
- Mycophenolate mofetil	37
- Azathioprine	9
- Cyclosporine	1
- Methotrexate	1
Vitamin D supplement (Ergocalciferol)	
- Intake (n)	87
- Dose, international unit/day, median (IQR)	2166.66 (1333.33–2666.67)
Chronic kidney disease^a^ (n)	32

Total sample size = 100, n = %

^a^Defined as glomerular filtration rate < 90 ml/min/1.73m^2^

### Characteristics of sunscreen use and the frequency of sunscreen application

At the first assessment, 79 (79%) of patients reported using sunscreen. The most commonly reported reason for not using sunscreen was thinking it was unnecessary. At the second assessment, the percentage of patients who used sunscreen significantly increased to 92%. Three patients changed the reason for not using sunscreen from thinking it was unnecessary to its stickiness, making the latter the most commonly reported reason for not using sunscreen instead.

Regarding the characteristics of sunscreen use, the majority of patients (77%) reported using sunscreen with a sunburn protection factor (SPF) ≥ 30 at the first assessment and significantly increased to 91% at the second assessment. Most patients used a sunscreen with protection grade of ultraviolet A (PA) +  +  + . A minority of patients used water-resistant sunscreen and lip balm with sunscreen but the proportion of patients who used lip balm with sunscreen was significantly increased at the second assessment. Concerning the frequency of sunscreen application, 52% of patients applied sunscreen every day, and this proportion significantly increased to 65% at the second assessment. At both the first and second assessments, a minority of patients reported reapplying sunscreen every 2 h when sweating or engaging in outdoor activities. Details specific to the characteristics of sunscreen use and the frequency of sunscreen application are presented in Table 2.

**Table 2:** Characteristics of sunscreen use and the frequency of sunscreen application

Characteristics	First assessment	Second assessment	*p*-value
Using sunscreen (n)	79 (79%)	92 (92%)	0.002
Reason(s) for not using sunscreen (n)^a^			-
- It is unnecessary	13 (13%)	2 (2%)	
- It is sticky/don’t like the feel/texture	7 (7%)	5 (5%)	
- It is expensive	2 (2%)	1 (1%)	
- Using whitening cream instead	2 (2%)	1 (1%)	
- It takes too much time	1 (1%)	0 (0%)	
SPF level of sunscreen ≥ 30 (n)	77 (77%)	91 (91%)	0.001
PA level of sunscreen (n)			-
- +	3 (3%)	0 (0%)	
- + +	4 (4%)	7 (7%)	
- + + +	63 (63%)	74 (74%)	
- + + + +	9 (9%)	11 (11%)	
Water resistant sunscreen (n)	34 (34%)	39 (39%)	0.42
Frequency of sunscreen application (n)			
Every day	52 (52%)	65 (65%)	0.02
Not every day but applied			
- When going outside/doing outdoor activities	18 (18%)	23 (23%)	-
- On sunny days	16 (16%)	25 (25%)	-
Reapply sunscreen when sweating/doing outdoor activities every 2 h (n)	13 (13%)	22 (22%)	0.12
Using lip balm with sunscreen (n)	23 (23%)	42 (42%)	0.001

Total sample size = 100, *n* = %

^a^A patient may have more than one reason for not using sunscreen

*PA* Protection grade of ultraviolet A, *SPF* Sunburn protection factor

### The amount of sunscreen used and area of sunscreen coverage

The amount of sunscreen applied to the face was ≥ 2 fingertip units in 32% of patients, and that proportion significantly increased to 71% at the second assessment. The facial anatomical sites to which sunscreen was applied least often were the ears, hairline and periorbital area in descending order. These numbers were not significantly increased at the second assessment. Regarding the sun exposed areas of the body, the least and second least anatomical body sites to which sunscreen was applied were the dorsum of the feet and dorsum of the hands, respectively. Sunscreen application on the dorsum of the feet was not significantly increased at the second assessment. The median of body skin surface area covered by sunscreen was 43% (IQR 2–47) at the first assessment, and was significantly increased to 45% (IQR 43–50) at the second assessment. Details specific to the amount of sunscreen used and area of sunscreen coverage as presented in Table 3.

**Table 3:** The amount of sunscreen used and area of sunscreen coverage

Sunscreen application	First assessment	Second assessment	*p-*value
**Amount of sunscreen applied to the face (n)**			
- ≥ 2 fingertip units	34	71	< 0.001
**Sunscreen coverage by anatomical facial site (n)**			
- Hairline	12	12	> 0.99
- Forehead	68	89	< 0.001
- Ears	4	11	0.07
- Periorbital	21	32	0.06
- Nose	65	88	< 0.001
- Cheeks	72	90	< 0.001
- Nasolabial	70	89	< 0.001
- Perioral	68	88	< 0.001
**Sunscreen coverage of the sun exposed areas of the body (n)**			
- Neck	38	66	< 0.001
- Forearm	72	84	0.02
- Dorsum of hands	17	29	0.008
- Legs	70	89	< 0.001
- Dorsum of feet	12	20	0.06
**Percentage of skin surface covered, median (IQR)**	43 (2–47)	45 (43–50)	< 0.001

Total sample size = 100, *n* = %

### Sun Protection Habit Index (SPHI)

The most commonly practiced sun protection behavior was wearing sunscreen, and the least often practiced sun protection behavior was wearing sun glasses. These two behaviors had not significantly increased at the second assessment. The patients who rarely/never wore sunglasses had significantly lower household income than the rest of the patients at the second assessment (10,000 [IQR 5,500- 20,000] vs 22,500([IQR13,325–71,500], *p*-value = 0.028). SPHI was significantly higher at the second assessment as shown in Table 4. The duration of SLE did not significantly correlate with the SPHI (*ρ* = 0.03, *p* = 0.79).

**Table 4:** Sun Protection Habit Index

Sun protection behaviors^a^	First assessment	Second assessment	*p*-value
Wear sunscreen, median (IQR)	3 (2–4)	3 (2–4)	0.93
Wear a shirt with sleeves, median (IQR)	2 (2–3)	3 (2–3)	< 0.001
Wear a hat, median (IQR)	2 (1–2)	2 (2–3)	< 0.001
Stay in the shade or under an umbrella, median (IQR)	2 (1–2)	2 (2–3)	< 0.001
Wear sunglasses, median (IQR)	1 (1–1)	1 (1–1)	0.75
SPHI, median (IQR)^b^	2 (1.7–2.4)	2.2 (2–2.6)	< 0.001

^a^Each line item reflects the median of 4-point Likert scale scoring, ranging from 1 (rarely or never) to 4 (always)

^b^The median of the average of 4-point Likert scale scoring of all sun protection behaviors

*SPHI *Sun Protection Habit Index

### Time of day, and the activities engaged in during sun exposure

During 6:00 am to 10:00 am, the most frequently reported activities engaged in during sun exposure were travelling to school and attending the national flag ceremony. During 10:01 am to 4:00 pm, the most often reported activities were playing with friends and walking to the cafeteria. During 4.01 pm to 6:00 pm, the most commonly reported activities were traveling home from school and playing with friends/relatives. More details specific to the activities engaged in during sun exposure are shown in Table 5

**Table 5:** Time of sun exposure during the day and the activities engaged in during sun exposure

Time and activities^a^	First Assessment (n)	Second assessment (n)	*p-*value
**6:00 am to 10:00 am**	71	60	0.09
- Traveling to school	34	23	
- Attending the morning national flag ceremony	14	11	
- Going to the market/grocery store	11	14	
- Changing classroom to another building	10	6	
- Playing with friends/relatives	7	2	
- Exercise	6	4	
- Helping with outdoor chores at home	5	10	
**10:01 am to 4:00 pm**	52	56	0.64
- Playing with friends	21	26	
- Walking to the cafeteria	21	24	
- Changing classroom to another building	16	9	
- Exercise	6	4	
**4.01 pm to 6:00 pm**	78	69	0.12
- Traveling home from school	32	25	
- Playing with friends/relatives	23	30	
- Exercise	19	11	
- Going to the market/grocery store	8	11	
- Helping with outdoor chores at home	8	1	

Total sample size = 100, *n* = %

^a^A patient may have engaged in more than one activity during the time period

The number of patients exposed to the sun was not significantly decreased in all time periods when compared between the first and second assessments.

### Association between photoprotection and SLE disease activity

The SLEDAI-2 K and CLASI-activity scores were significantly decreased at the second assessment (Table 6; *p* = 0.002 and 0.003 respectively). However, a multivariate logistic regression model revealed that this was caused by the effect of the changes in medication doses (Adjusted odds ratio for medication doses = 4.06 (95% CI 1.29–15.52); *p* = 0.024). We found no significant correlation between SPHI and SLE disease activity (Table S1).

**Table 6:** SLE disease activity and serum vitamin D level compared between the first and second assessments

Parameter	First assessment	Second assessment	*p*-value
SLEDAI-2 K, median (IQR)	4 (2–8)	4 (2–6)	0.002
CLASI-activity score, median (IQR)	2 (0–2)	0 (0–0)	0.003
CLASI-damage score, median (IQR)	0 (0–0)	0 (0–0)	0.61
Vitamin D level, ng/ml, mean (SD)	25.9 (7.6)	27.9 (6.9)	0.06

*CLASI* Cutaneous Lupus Erythematosus Disease Area and Severity Index, *SLEDAI-2 K* Systemic Lupus Erythematosus Disease Activity Index 2000, *SPHI* Sun Protection Habits Index

### Effect of photoprotection on serum vitamin D level

There were no statistically significant differences in the serum vitamin D level between the first and second assessments as shown in Table 6. There were no significant correlations between the SPHI and serum vitamin D level (Table S1).

## Discussion

Although the relationship between UV radiation and SLE is well evidenced [1], photoprotection could be inadequately practiced. To improve our understanding of the photoprotection of SLE children, we enrolled 100 SLE patients aged ≤ 18 years. The photoprotection questionnaire and status of disease activity were collected and compared from baseline to the ≥ 3-month follow-up time point. Photoprotection education was provided. We found that the majority of patients were practicing photoprotection but not accurately in some areas. Education on photoprotection was effective There were no associations between photoprotection and SLE disease activity or serum vitamin D level.

Compared to the first assessment where 79% of patients used sunscreen, our second assessment showed an improvement of 92% rate of using sunscreen. The rate of sunscreen use is higher compared to the adult studies from Abdul Kadir et. al (52.3%) [7] and Chanprapaph et al.(63.5%) [25] but is approximately close to another study in children in Thailand (95.8%) [26]. This may be because Thai families place value on family connections and close parent–child relationships. Children tend to be more dependent on parents, especially for health and self-care. Thus, our patients were constantly reminded by their parents to use sunscreen or even had their parents apply sunscreen for them.

At the first assessment, the main reason for not applying sunscreen was that our patient thought it was unnecessary. These patients thought that if they stayed indoors or used physical photoprotection then they did not need to apply sunscreen. This underlines the need to counsel patients with SLE that optimal photoprotection requires daily sunscreen application. At the second assessment, the main reason for not using sunscreen switched from thinking it was unnecessary to disliking its texture. This reason is in line with a study from Malaysia, a tropical country with hot climate that makes sunscreen greasier, similar to Thailand [7]. This is in contrast with a study from the USA in which the inherent features of sunscreens was the third common reason for not using sunscreen [6]. This reason can be diminished by improving the texture of sunscreen.

The recommended amount of sunscreen use is 2 mg/cm [2] which is difficult to achieve in real life practice. As a result, a fingertip unit or teaspoon unit has been recommended [23, 27]. We found that at the first assessment, a majority of the patients applied sunscreen less than the recommended amount, which is in line with previous studies in SLE patients [7, 26] and in the general population [28, 29]. An inadequate amount of applied sunscreen significantly decreases its efficacy [30]. Therefore, the patients need to be instructed as to the correct application and amount of sunscreen.

The facial anatomical sites to which sunscreen was covered least often were the ears, hairline and periorbital areas. These findings are in line with the study of Loesch et al. [31] In the study, the authors stated that the reasons that the subjects did not apply sunscreen at the hairline and periorbital areas as it would grease their hair and sting their eyes. These reasons could be similar for our patients and such obstacles could be addressed if the sunscreen texture were improved. Interestingly, our study was conducted during the COVID-19 pandemic when wearing a mask was mandatory. However, the majority of patients still applied sunscreen at under the mask areas. The least and second least anatomical body sites to which sunscreen was covered were the dorsum of the feet and dorsum of the hands, respectively. These mentioned areas should be specifically emphasized to apply sunscreen on. Sunscreen application on the dorsum of the feet was not significantly increased at the second assessment. This was likely due to Thai students being required to wear socks as a part of their school uniform. Therefore, patients were unlikely to consider this area to be exposed to the sun.

The least often practiced sun protection behavior was wearing sunglasses which was not improved at the second assessment after education on photoprotection. Although Thailand is a middle-income country, the distribution of poverty is uneven across the regions with the poverty rate in the Northeast almost double the national level, which is where our hospital is based [32]. Standardized sunglasses can be costly and are not affordable for our patients. As demonstrated in our study, after patients were educated that wearing sunglasses was one aspect of photoprotection, the patients who continued to rarely/never wear sunglasses as shown at the second assessment had significantly lower household income than the rest of the patients. This indicates that education could not improve behaviors around wearing sunglasses if the family could still not afford them. This socioeconomic reason was confirmed by another study from Thailand which found that sunglasses were more frequently used in patients with a higher family income [26].

During 6:00 am to 10:00 am, the most frequently reported activities engaged in during sun exposure were travelling to school and attending the national flag ceremony. The majority of our patients travelled to school by motorcycles which is the most used vehicle in Thailand as it is less-expensive and more convenient compare to other automobiles. Unfortunately, only 43% of people in Thailand wear helmets—52% of drivers and 20% of passengers [33]. Moreover, child motorcycle helmets with UV-resistant visors, are simply not affordable, as 43% of our patients had an average household income less than the sustainable living wage [34]. As a result, our patients are at risk of both head injury and UV exposure. In Thailand, there is a unique activity engaging in sun exposure which is the morning national flag ceremony. The students will line up at the school grounds, which are mostly outdoor and uncovered to sing national anthems. After that, the teachers will inform about important issues. The activity takes about 30 min. The activity starts at 8 am but the UV index can be not low during a sunny day. In the authors’ experiences even though our patients know to avoid this activity, they still would like to attend as they don’t want to be seen as weak students. Therefore, it is important that the physicians should formally inform their patients’ schools regarding the need to avoid sun exposure.

The most often reported activities during 10:01 am to 4:00 pm, which is during the peak UV index, were playing with friends and walking to the cafeterias. Playing with friends is crucial in enhancing development. However, school facilities may not be feasible for indoor recreation activities. In addition, some schools in Thailand have cafeterias in different buildings. Thus, these patients need to use physical photoprotection i.e. hat, umbrella or long-sleeve shirts. In the authors’ experiences, these are difficult to practice especially in our teenage patients as it makes them feel alienated from their peers.

Thailand is an agricultural country in which farming household plays an important role. Some of our patients had to help with outdoor chores such as helping in the farm fields, taking care of cattle, rubber tapping and going to the market/grocery store. Fortunately, most of the outdoor chores are done before or after school when the intensity of UV radiation is lower.

The number of patients exposed to the sun was not significantly decreased in all time periods at the second assessment. This was due to socioeconomic status which could not be changed by education alone i.e. the patients still had to travel by motorcycle and help with outdoor chores.

Education on photoprotection was effective in improving photoprotection practices, including applying an adequate amount of sunscreen and using lip balm with sunscreen, which were behaviors practiced among only a minority of our patients at the first assessment. Moreover, the reason for not using sunscreen was changed from thinking it was unnecessary to disliking its texture at the second assessment. Therefore, these aspects should be vigorously emphasized. Although using water resistant sunscreen, reapplying sunscreen when sweating/doing outdoor activities, applying sunscreen to commonly missed areas, wearing sunglasses, and avoiding sun exposure were not improved by the second assessment, these areas should still be emphasized, esp. in populations with different contexts from our patients, e.g., people who have better access to a pleasant texture of sunscreen or affordable water-resistant sunscreen, people with more flexible break times to reapply sunscreen, those with different or no school uniforms, or those of a higher socioeconomic status. Written and verbal patient education on photoprotection should be given at the diagnosis point and periodically reviewed. As at the first diagnosis point, the seriousness of SLE and information regarding the disease can be overwhelming. Physicians should update their knowledge regarding photoprotection as there are still some knowledge gaps even in subspecialists [35].

There was no direct association between photoprotection and SLE disease activity which is similar to a previous study [7] in which good photoprotection practice was not a predictor of disease activity. Our finding is in contrast with Vilá et al. [8] who found that patients that regularly used sunscreen had significantly lower renal involvement, thrombocytopenia, hospitalizations, and requirement of cyclophosphamide treatment than patients who did not use sunscreen. However, the study was a cross-sectional study and evaluated clinical outcomes by reviewing medical records. One of the main reasons behind this negative finding is that UV has more direct effects on cutaneous lupus than it does on systemic disease [36]. In addition, only one-third of our patients had cutaneous lupus, and their CLASI scores were low to begin with. Moreover, the other factors could affect the disease activity of SLE and not UV radiation exposure alone. These factors include but are not limited to medications [37], infection [38], tobacco smoke and diet [39].

We found no effect of intensification of photoprotection on serum vitamin D levels, which contradicts previous studies [14, 40]. The reason could be that we routinely check the serum vitamin D levels at the diagnosis of SLE and every 3 months until levels are normal. Therefore, only a minority of our patients had an abnormal serum vitamin D level. Moreover, the vitamin D pathway requires UV-B exposure in the skin, and SPF30 blocks 97% of it [41]. Seventy-nine percent of our patients used sunscreen of SPF30 and above on initial assessment. Thus, the change in vitamin D level was too small to reach statistical significance. Considering that photoprotection should be adequately practiced without a risk of vitamin D deficiency, monitoring serum vitamin D levels and vitamin D supplements should be routinely practiced as per recommendations [42, 43].

This study has some mentionable limitations**.** Patient responses to the items in the study questionnaire may not accurately reflect the real-life photoprotection since it is known that questionnaire respondents are sometimes inclined toward responding more favorably than is actually true to avoid a loss of credibility and to please their physicians. However, study patients completed the questionnaire privately apart from their subspecialists, and the completed questionnaire was immediately collected by a research assistant. Moreover, the patient questionnaire responses were not evaluated by the research team until the end of the study to reduce the probability of assessment bias. Importantly, the fact that some patients reported inadequate photoprotection on both the baseline and follow-up the photoprotection questionnaires suggests the veracity of the answers to the questionnaire items.

The strengths of this study are the comprehensive evaluation of the practical aspects of photoprotection. The study was also prospectively conducted in a tropical country with a high UV index all year round [44] and a unique culture and socioeconomic structure. In addition, our study was conducted in the pediatric population in which the data is still scarce. To the best of our knowledge, this is the first study that prospectively conduct to evaluate photoprotection and the effect of education in pediatric SLE patients.

## Conclusion

The majority of our patients practiced photoprotection accurately and adequately. Education on photoprotection was effective in improving photoprotection practices, including applying an adequate amount of sunscreen and using lip balm with sunscreen, which were practiced in a minority of our patients at the first assessment. Although using water resistant sunscreen, reapplying sunscreen when sweating/doing outdoor activities, applying sunscreen to commonly missed areas, wearing sunglasses, and avoiding sun exposure were not improved by the second assessment, these areas should still be emphasized in a population who may have a different context or background from our patients. There were no direct associations between photoprotection and disease activity or serum vitamin D level.

### Supplementary Information

**Additional file 1: Supplementary Table S1. **Correlation between parameters.

## Data Availability

The datasets used and/or analysed during the current study are available from the corresponding author on reasonable request.
