# Supplementary material for: Accuracy and adequacy of photoprotection in pediatric systemic lupus erythematosus patients, and the effect of education on photoprotection: a prospective study
Source: Pediatr Rheumatol Online J. 2023 Oct 17;21:123. doi: 10.1186/s12969-023-00901-z (PMC10583389; doi:10.1186/s12969-023-00901-z)
Supplement: Supplementary file 1 — Additional file 1: Supplementary Table S1. Correlation between parameters. [file 12969_2023_901_MOESM1_ESM.docx]

| **Parameters** | **ρ** | ***p*-value** |
| --- | --- | --- |
| SPHI and SLEDAI-2K at the first assessment | – 0.07 | 0.49 |
| SPHI and SLEDAI-2K at the second assessment | – 0.08 | 0.41 |
| SPHI and CLASI-activity score at the first assessment | 0.06 | 0.56 |
| SPHI and CLASI-activity score at the second assessment | – 0.06 | 0.08 |
| SPHI and CLASI-damage score at the first assessment | 0.03 | 0.80 |
| SPHI and CLASI-damage score at the second assessment | – 0.07 | 0.47 |
| SPHI and vitamin D level at the first assessment | – 0.05 | 0.64 |
| SPHI and vitamin D level at the second assessment | – 0.13 | 0.19 |

***Supplementary Table S1.* Correlation between parameters.**

CLASI: Cutaneous Lupus Erythematosus Disease Area and Severity Index; SLEDAI-2K: Systemic Lupus Erythematosus Disease Activity Index 2000; SPHI: Sun Protection Habits Index
